# Remodeling and Alteration of the Gut Microbial Community in *Suncus murinus* Receiving Fecal Microbiota Transplantations from Mice with High-Fat Diet-Induced Obesity

**DOI:** 10.3390/nu18213626

**Published:** 2026-10-31

**Authors:** Yuhao Zeng, Mingshou Zhang, Ke Ren, Ting Yang, Jun Li, Shuang-Qin Yi

**Affiliations:** 1 Department of Frontier Health Sciences, Graduate School of Human Health Sciences, Tokyo Metropolitan University, Tokyo 116-8551, Japan; 2 School of Public Health and Health Management, Gannan Medical University, Ganzhou 341000, China; 3 Faculty of Physical Education, Qu Jing Normal University, Qujing 655000, China; 4 Division of Anatomical Science, Department of Functional Morphology, Nihon University School of Medicine, Tokyo 173-8610, Japan; 5 State Key Laboratory of Oncogenes and Related Genes, Shanghai Cancer Institute, Ren Ji Hospital, School of Medicine, Shanghai Jiao Tong University, Shanghai 200240, China

**Keywords:** high-fat diet, fecal microbiota transplantation, host filtering, microbiota engraftment, obesity resistance, *Suncus murinus*

## Abstract

Background: Obesity-associated gut microbiota can transmit metabolic traits in permissive recipients; however, microbial engraftment following fecal microbiota transplantation (FMT) is also constrained by the recipient’s intestinal environment. *Suncus murinus* (*S. murinus*) is an insectivorous and naturally obesity-resistant species with a distinctive gut ecology, making it a potential model for examining recipient filtering during cross-species FMT. Objectives: To determine whether cecal microbiota from high-fat diet (HFD)-fed mice induces persistent donor diet-specific microbial and host phenotypes in *S. murinus* relative to microbiota from normal chow diet (NCD)-fed donors. Methods: *S. murinus* recipients (*n* = 12) were randomly allocated to high-fat diet-recipient (HFD-r) and normal chow diet-recipient (NCD-r) groups (*n* = 6 per group). All recipients received the same normal trout diet throughout the study. The recipients were administered supernatants of donor cecal microbiota via oral gavage for three consecutive days. A 16S rRNA gene sequencing analysis was performed on fecal samples collected before antibiotic treatment and at weeks 4 and 8 post-FMT. Longitudinal microbial outcomes were analyzed using common baseline-constrained longitudinal models, restricted PERMANOVA, and false discovery rate correction. Results: Bacillota was the predominant phylum in both the HFD-r and NCD-r groups. The most consistent taxonomic response was a shared decrease in Campylobacterota and its major constituent genus Helicobacter after treatment, particularly at 4 weeks, whereas no robust phylum- or genus-level differences were detected between the HFD-r and NCD-r groups. Observed ASV richness and Faith’s phylogenetic diversity did not differ significantly among the groups. Principal coordinate analyses showed extensive overlap, and a restricted PERMANOVA detected no significant separation between the HFD-r and NCD-r groups. Histological examination revealed crown-like arrangements and heterogeneous adipocyte morphology, and variation in villus and crypt appearance in representative recipient sections. Conclusions: No significant differences in fecal microbial composition, body weight, or glucose tolerance were detected between the HFD-r and NCD-r groups. Recipient-associated ecological constraints remain a possible explanation requiring direct assessment of donor engraftment. Further work is needed to evaluate *S. murinus* as a model for investigating recipient-side constraints on microbiota colonization.

## 1. Introduction

Obesity has increased worldwide and is a major risk factor for cardiometabolic disease and cancer. Among the multiple drivers of obesity, shifts toward energy-dense dietary patterns, particularly the increased consumption of high-fat diets (HFDs), have garnered considerable attention. Accordingly, HFD feeding is widely used to model diet-induced obesity and associated metabolic disorders in rodents [1,2,3].

Accumulating evidence indicates that gut microbiota are closely linked to host energy balance and inflammatory tone. Obesity and HFD exposure are often associated with shifts in the gut microbial community structure and function, and early studies have proposed that such shifts may increase energy harvest from the diet and contribute to adiposity [4,5,6]. Diet can also remodel the gut microbiome, sometimes independently of obesity status, highlighting the importance of dietary context when interpreting microbiome–phenotype associations [7].

Fecal microbiota transplantation (FMT) and gnotobiotic approaches have been instrumental in moving beyond correlation to test causality. The transfer of obesity-associated microbiota has been shown to transmit metabolic phenotypes in mice, while transplantation studies using donors after bariatric procedures or with distinct metabolic states further support a functional role of microbiota in shaping host metabolism [8,9,10].

A key limitation in interpreting FMT outcomes is that engraftment is not a simple donor overwrite process; actual recipient ecology can strongly constrain which donor strains establish and persist. Strain-resolved studies have shown variable and often incomplete donor strain engraftment, with outcomes influenced by the recipient’s baseline microbiota state, ecological compatibility, and pretreatment regimens [11,12,13,14]. This framework suggests that host-specific intestinal environments can attenuate donor-associated signatures in fecal taxonomic profiles, even when biologically meaningful host responses occur. Recent studies have highlighted the importance of distinguishing donor engraftment from apparent post-FMT detection [15] and have described cohesive groups of donor species that engraft together while retaining their relative composition [16].

The house musk shrew, *Suncus murinus* (*S. murinus*), is an insectivore with a markedly simple gastrointestinal tract that lacks prominent fermentative chambers (e.g., cecum), implying nutritional physiology and microbial habitats distinct from those of common rodent models. Consistent with these constraints, *S. murinus* has been reported to harbor a Bacillota-rich gut microbiota with a comparatively low representation of Bacteroidetes [17]. Our recent research has shown that *S. murinus* possesses natural obesity-resistant properties, and when used as an FMT donor, its gut microbiota can reshape the gut ecology of diet-induced obese mice [18,19]. *S. murinus* was selected as the recipient species, as our previous research revealed that it possesses obesity resistance and distinctive intestinal anatomy and microbiota, providing a physiological background different from conventional mouse recipients [17,18,19].

However, it remains unclear whether microbiota derived from HFD-induced obese mice can establish and retain their donor diet-associated characteristics in *S. murinus* and whether these microorganisms can induce corresponding obesity-related host responses. Alternatively, the recipient-specific intestinal environment of *S. murinus* may constrain the establishment of donor microbiota and cause the microbial communities and host phenotypes to converge, irrespective of the donor diet. Therefore, the aim of the present study was to determine whether cecal microbiota derived from HFD-fed and normal-chow diet (NCD)-fed mice produce persistent differences in the gut microbial composition and obesity-related phenotypes of *S. murinus*. In this study, *S. murinus* received cecal microbiota from C57BL/6NCrSIc mice fed either an HFD or NCD. Fecal bacterial communities were analyzed at 4 and 8 weeks after FMT.

## 2. Materials and Methods

### 2.1. Animals

All animal experiments were approved by the Institutional Animal Care and Use Committee of Tokyo Metropolitan University and conducted in accordance with the National Research Council’s Guide for the Care and Use of Laboratory Animals (A7-009). Based on breeding availability and our previous FMT study [19], *S. murinus* recipients (*n* = 6 per group) were used without an priori sample-size or power calculation. The study used 12 mouse donors and 12 *S. murinus* recipients, with six animals per group in each species and one donor paired with each recipient. Repeated fecal sampling of the same recipients at three time points avoided separate cohorts for each time point, consistent with the Reduction component of the 3Rs.

### 2.2. C57BL/6NCrSIc Mice

As donors of FMT, specific-pathogen-free C57BL/6NCrSIc mice (male, *n* = 12, 6 weeks old) obtained from Sankyo Labo Service Corporation, Inc. (Tokyo, Japan) were randomly divided equally into HFD and NCD groups (*n* = 6 per group) and housed in high-efficiency, particulate air-filtered cages with ad libitum access to food and water. All mice were maintained on a 12-h light/dark cycle at 25 ± 2 °C with humidity controlled at 50% ± 5% in the Functional Morphology Laboratory, Department of Frontier Health Sciences, Tokyo Metropolitan University (Tokyo, Japan). The HFD group was fed a high-fat diet (60 kcal% fat; D12492; Research Diets Inc., New Brunswick, NJ, USA). The NCD group was fed a normal chow diet (NCD, 3.9% crude fat by weight; Labo MR Stock; Nosan Corporation, Yokohama, Japan). All food was replaced every 2 days to keep it fresh and palatable. Feeding continued until the mice in the HFD group reached a body condition score (BCS) of 5 [20]. The body weight of each donor mouse was measured weekly throughout the 8-week feeding period. All six donor mice in the HFD group were co-housed in one cage, and all six donor mice in the NCD group were co-housed in a separate cage. The two cages were maintained in different compartments to minimize the cross-transmission of intestinal microbiota between the diet groups. Thus, donor diet was confounded with cage and compartment. D12492 is a purified diet, whereas Labo MR Stock is grain-based chow; the diets therefore differ in components other than fat.

### 2.3. S. murinus

Male *S. murinus* (*n* = 12, 8–10 weeks old), obtained from a closed breeding colony (JIc: KAT-c) at our laboratory, were used as recipients for FMT experiments in this study. All recipient animals were housed in polycarbonate cages with high-efficiency particulate air filtration, sterilized bedding, and *ad libitum* access to food and water, under a 12-h light/dark cycle at 28 ± 2 °C with 50% ± 5% relative humidity. All recipients received the same normal trout diet (NTD) used by Zhang et al. [19] before and after FMT, without adaptation to HFD or NCD. In our colony, these animals accepted NTD but refused the alternative diets offered. The pellets consisted of 45.0% protein, 4.0% fat, 3.0% fiber, 15.0% ash, and 26.2% complex carbohydrates (Oriental Yeast Co., Ltd. Bioindustry Division, Chiba, Japan), and the metabolizable energy content was 357 kcal/100 g [21].

**Figure 1:**
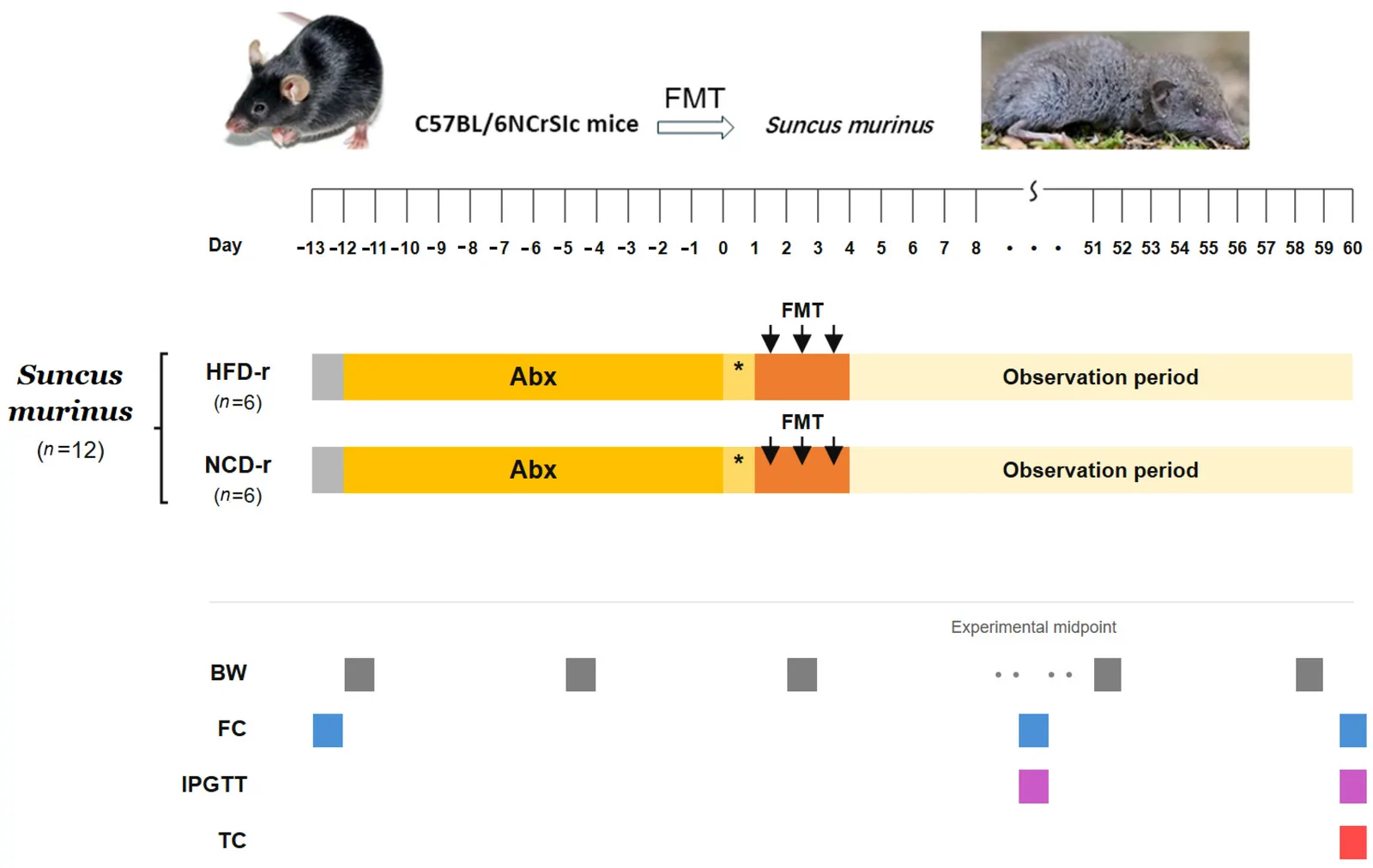
Experimental design and sampling schedule for *S. murinus* recipients. HFD-r, recipients of microbiota from HFD-fed mice; NCD-r, recipients of microbiota from NCD-fed mice. Abx, antibiotic treatment; BW, body weight measurement; FC, fecal sample collection; FMT, fecal microbiota transplantation; IPGTT, intraperitoneal glucose tolerance test; TC, tissue collection; *, one-day interval between Abx treatment and FMT.

Twelve *S. murinus* individuals were randomly assigned to either the high-fat diet recipient (HFD-r) or the normal chow diet recipient (NCD-r) group (*n* = 6 per group); they received fecal microbiota transplants from mice donors fed a high-fat diet and a normal-chow diet, respectively. The grouping and numbering of the animals were carried out by different researchers using a blinded approach. After 12 days of antibiotic treatment and a 1-day withdrawal period, the recipients received FMT once daily for three consecutive days. FMT preparations were derived from the cecal contents of the corresponding donor mice. Fecal samples were collected prior to antibiotic treatment and at weeks 4 and 8 post-FMT (labeled as HFD-r/4w, HFD-r/8w, and NCD-r/4w, NCD-r/8w, respectively, in the figures); the 12 samples collected prior to antibiotic administration served as the common pretreatment baseline (CON) for group-level microbiome analyses. Additionally, body weight measurements, intraperitoneal glucose tolerance tests, and tissue collection were performed in each group at the scheduled time points (Figure 1).

### 2.4. Experimental Design and Procedure

As shown in Figure 1, fecal samples were collected longitudinally from each recipient at three time points: before antibiotic treatment (baseline), at week 4 post-FMT, and at week 8 post-FMT. For group-level microbiome analyses and visualizations, all 12 baseline samples were assigned to a common baseline category designated CON, irrespective of the subsequent donor group allocation. Post-FMT samples were categorized as HFD-r/4w, HFD-r/8w, NCD-r/4w, and NCD-r/8w (*n* = 6 per category). The CON consisted of 12 individual baseline samples and was used as a common pretreatment reference rather than representing a separate untreated control cohort. Individual animal identities were retained at the three sampling points. No concurrent antibiotic-only, sham-FMT, or untreated groups were included. The study compared the two donor sources under common recipient feeding and pretreatment conditions.

Both groups received 12 days of antibiotic treatment, followed by a 1-day drug withdrawal period. An antibiotic cocktail—including ampicillin (1.0 g/L; Nacalai Tesque, Kyoto, Japan), vancomycin (0.5 g/L; Shionogi, Osaka, Japan), neomycin (1.0 g/L; Nacalai Tesque, Kyoto, Japan), metronidazole (1.0 g/L; Nacalai Tesque, Kyoto, Japan) [21], and amphotericin B (0.01 g/L, FUJIFILM Wako Pure Chemical Corporation, Osaka, Japan) [22]—and sucrose (3%, FUJIFILM Wako Pure Chemical Corporation, Osaka, Japan) [23,24] were added to drinking water.

### 2.5. Fecal Microbiota Transplantation

FMT was performed according to the protocol established in our previous study [21]. All surgical instruments were aseptically sterilized before transplantation surgery to collect fecal content from the donor animal. Age- and sex-matched donor animals (C57BL/6NCrSIc mice) were deeply anesthetized under inhalation anesthesia with isoflurane (concentration 5.0%, *v*/*v*) using a continuous inhalation anesthesia machine (SN-487-OT Air, WAKENYAKU CO., Ltd., Kyoto, Japan). The abdominal skin was disinfected with iodine, and a midline abdominal incision was made. The cecum was cut open under sterile conditions, and approximately 100 mg of luminal content was carefully collected and transferred into a 5 mL sterile microtube to avoid external contamination. The sample was resuspended in 1 mL of sterile phosphate-buffered saline (PBS; pH 7.2) containing 10% glycerol and 0.05% L-cysteine [25] in a 5 mL sterile microtube, mixed with a vortex mixer for 10 s, and centrifuged at 805× *g* for 3 min at 4 °C to separate the supernatant that contained most of the microbiota from the solid fecal matter. Approximately 600 µL of the supernatant was removed and divided into three equal parts (200 µL/tube) and then stored at −80 °C in a freezer for transplantation. Two hundred microliters of intestinal content supernatant were administered via oral gavage for three days. Both groups were fed sterile water after transplantation until the end of the experiment. After sample collection from the donor animals, the donor animals were euthanized using an overdose of anesthesia. All three aliquots administered to each recipient originated from the same paired donor. No deaths or infectious complications from FMT were observed in this study.

### 2.6. Body Weight and Serum Collection and Processing

The body weight was measured once a week. The first two measurements were obtained during antibiotic pretreatment before FMT; FMT was marked at the third measurement, followed by post-FMT measurements.

Serum samples were collected at three time points: before antibiotic treatment and at 4 and 8 weeks after FMT. For the first and second collections, approximately 100 µL of blood was obtained via external jugular vein puncture. For the final sampling, approximately 100–200 µL of blood was directly collected from the left ventricle under deep anesthesia. All samples were centrifuged at 3000 rpm for 10 min at 4 °C, and the resulting serum was stored at −80 °C until further analysis.

### 2.7. Intraperitoneal Glucose Tolerance Test (IPGTT)

Glucose tolerance tests were performed at 4 and 8 weeks after FMT was performed. *S. murinus* were fasted overnight before testing. A glucose solution (2.0 g/kg body weight, 20% *w*/*v* in sterile PBS) was administered via intraperitoneal injection. Blood samples were collected from the tail vein at 0, 15, 30, 60, 90, and 120 min after glucose administration. Blood glucose levels were measured using a glucometer (Terumo Corporation, Tokyo, Japan). No pretreatment or concurrent untreated-control IPGTT was performed.

### 2.8. Extraction of DNA from Feces

Fresh fecal samples were collected at three time points: before antibiotic treatment and at 4 and 8 weeks after FMT. To obtain fresh feces, *S. murinus* individuals were gently suspended by the tail in an inverted position to stimulate immediate defecation. Approximately 100–150 mg of feces were collected, and the collected feces were immediately dissolved with reagents from the DNA Kit. Fecal DNA was extracted from the fecal samples using the ISOSPIN Fecal DNA Kit (Nippon Gene Co., Ltd., Toyama, Japan) according to the manufacturer’s protocols. All DNA samples were immediately stored at −80 °C before the next step [21].

### 2.9. Library

The V3–V4 region was amplified using Bakt_341F–805R primers. Library preparation used Herculase II Fusion DNA Polymerase (Agilent Technologies, Inc., Santa Clara, CA, USA) and the Nextera XT Index V2 Kit (Illumina, Inc., San Diego, CA, USA), following the Illumina 16S Metagenomic Sequencing Library Preparation protocol (Part No. 15044223 Rev. B). The project-specific Macrogen report identifies Illumina paired-end sequencing with a read length of 301 bp per end. Read-level quality control is described in Section 2.10.

### 2.10. Data Preprocessing and Amplicon Sequence Variant (ASV) Generation

After sequencing, Cutadapt (v3.2) [26] was used to remove adapter and primer sequences from the raw data. Additionally, forward and reverse reads were trimmed to 250 bp and 200 bp, respectively, using Cutadapt (v3.2). To generate ASVs, the reads were error-corrected, merged, and denoised using DADA2 (v1.18.0) [27]. Sequences with an expected error of ≥2 were excluded. Erroneous reads were denoised using an established error model. Following error correction, paired-end reads were merged by overlapping. Chimeric sequences were eliminated using the consensus method with the removeBimeraDenovo function in DADA2. ASVs with lengths shorter than 350 bp were filtered out using R (v4.0.3). Moreover, for a microbial community comparison analysis, normalization was performed using QIIME (v1.9) [28]. During this process, subsampling was conducted based on the sample with the lowest read count among all samples to ensure their comparability. The resulting ASVs were used for the downstream analysis.

### 2.11. Taxonomy Analysis and Community Diversity

Each ASV was aligned to the organism with the highest similarity in the corresponding Reference Database (NCBI_16S) using algorithms such as the Bayesian classifier (DADA2_v1.18.0, confidence value: 50) [29]. QIIME (v1.9.0) was used for the downstream ASV analysis. Alpha diversity metrics, such as the Shannon index, Gini–Simpson index, and PD whole tree, were calculated to represent species complexity within the individual samples. The observed ASV richness was also calculated. MAFFT (v7.475) [30] and FastTreeMP (v2.1.10) [31] were used to perform multiple alignments and construct the ASV phylogenetic tree. Beta diversity (differences in microbial community composition among samples) was calculated based on Bray–Curtis, Weighted UniFrac, and Unweighted UniFrac distances. Differences in microbial community composition among samples were visualized using PCoA and UPGMA trees.

### 2.12. Statistical Analysis

Longitudinal microbiome statistical analyses were performed in R (ver. 4.4.2), with individual recipients treated as experimental units. The 12 baseline samples were retained as individual observations and modeled as a common pre-FMT baseline (CON); they were not averaged into a single observation. Because recipients were randomly allocated before treatment and each animal was sampled at baseline and at 4 and 8 weeks, longitudinal outcomes were analyzed using a common baseline constrained longitudinal data analysis (cLDA) implemented as a linear mixed-effects model. The model contained a common baseline intercept and indicator terms for HFD-r/4W, HFD-r/8W, NCD-r/4W, and NCD-r/8W, with the animal ID included as a random intercept. Prespecified contrasts compared each post-FMT group with the CON, HFD-r with NCD-r at weeks 4 and 8, and the two donor groups across the post-FMT period.

For alpha diversity, Shannon diversity was analyzed without transformation, whereas observed ASV richness and Faith’s phylogenetic diversity were naturally log-transformed before cLDA. The same planned contrasts were applied to all three indices. Body weight was analyzed using a two-factor mixed-design repeated-measures ANOVA, with group and measurement occasion as factors and Greenhouse–Geisser correction for time and group-by-time effects. IPGTT outcomes were compared between the HFD-r and NCD-r groups using independent-samples *t*-tests. Histological findings were summarized descriptively without quantitative morphometry or blinded scoring. These outcomes were not included in the microbiome multiple-testing families; *p* < 0.05 was used for body weight and IPGTTs.

For phylum- and genus-level abundance testing, taxa were retained when they were detected in at least 20% of the samples and had an overall mean relative abundance of at least 0.1% within the corresponding taxonomic rank. The primary analysis used centered-log-ratio (CLR)-transformed rarefied counts after adding a pseudocount of 0.5 to each retained taxon [32]. Arcsine-square-root-transformed relative abundances were analyzed using the same cLDA model and contrast structure for sensitivity analysis. Findings supported by only one analytical scale were treated as exploratory rather than methodologically robust.

Beta diversity was assessed from Bray–Curtis, unweighted UniFrac, and weighted UniFrac distance matrices using restricted exact Permutational Multivariate Analysis of Variance (PERMANOVA) [33]. In the comparative analysis between the HFD-r and NCD-r groups at weeks 4 and 8, all 924 possible combinations of reassigning six animals to each donor group were used. Comparisons between post-FMT and baseline states were restricted to within-subject comparisons for each recipient animal, encompassing all 64 possible combinations of sample label permutations (baseline vs. post-FMT) across the six recipient pairs. An overall comparative analysis following FMT was conducted for the HFD-r and NCD-r groups based on subject-level centroids calculated from samples collected at weeks 4 and 8 in the full principal-coordinate space, retaining positive and negative eigenvalue components. The homogeneity of multivariate dispersion was examined using PERMDISP with spatial medians and full-model residual permutations under the corresponding allocation restrictions [34]. Except for the conventional upper-tail permutation tests used for PERMANOVA and PERMDISP, all *t*-tests and model-based contrasts were two-sided. Accordingly, *p*-values were adjusted using the Benjamini–Hochberg false discovery rate procedure within each prespecified family of alpha diversity or taxonomic rank contrasts [35]. For PERMANOVA, correction was applied jointly across 21 comparisons: nine between-group and 12 paired baseline-versus-follow-up comparisons. PERMDISP *p*-values are unadjusted. Adjusted *p*-values are reported as q-values, and q < 0.05 was considered to be statistically significant. The reported distance-based results were recalculated in Python (version 3.12.14) using NumPy and SciPy; exact *p*-values include the observed configuration (Supplementary Table S1).

## 3. Results

### 3.1. Body Weight Changes in Each Experimental Group

To compare the body weight between the two recipient groups, body weight was monitored throughout the experiment. The results showed no significant effects of group, measurement occasion, or their interaction on body weight (Supplementary Figure S1). The IPGTT curves at weeks 4 and 8 also showed comparable glucose responses, with no significant differences between the experimental groups.

### 3.2. Histological Analysis of Adipose Tissue and Small Intestine

**Figure 2:**
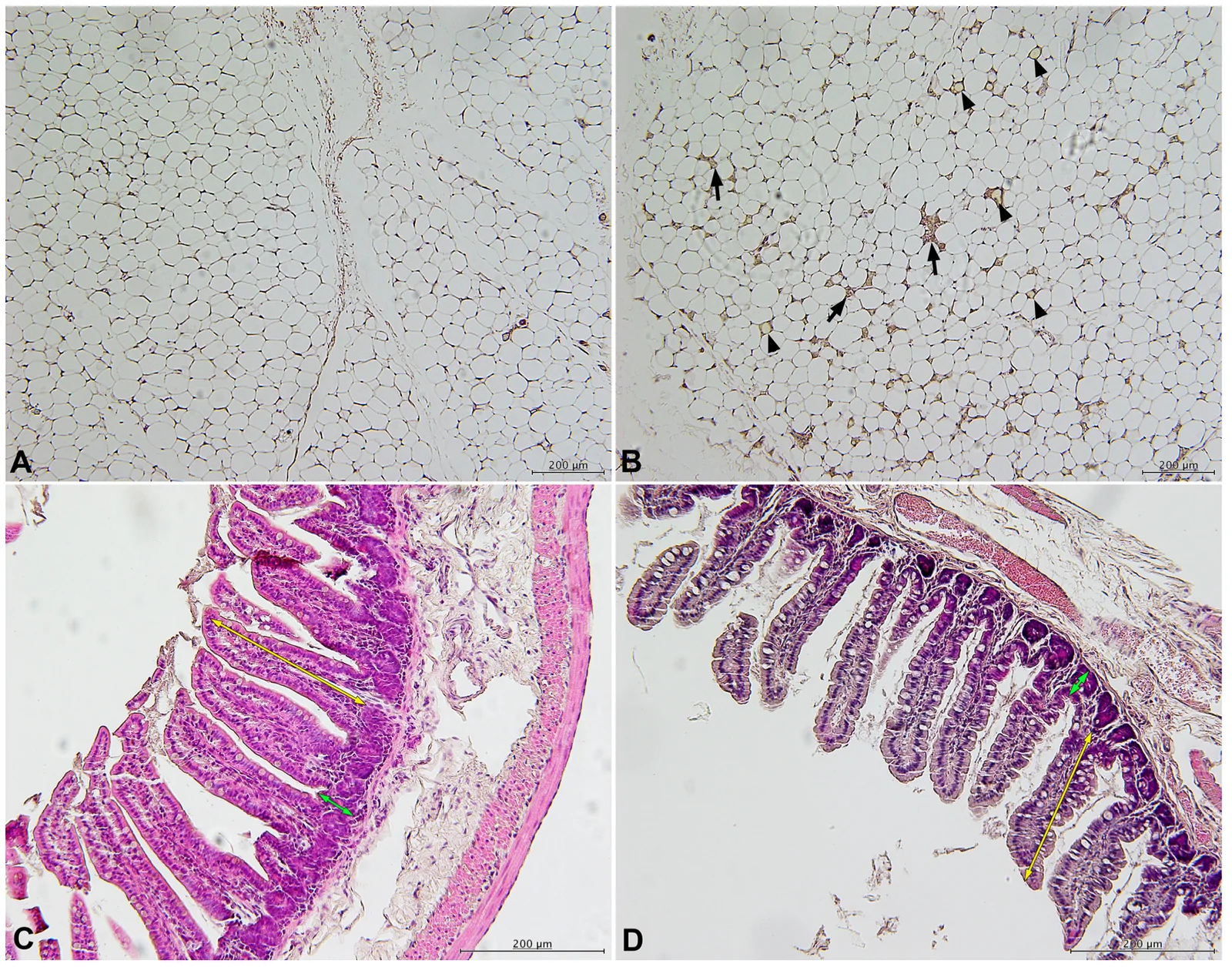
Serial sections of small intestinal and fat tissues from the NCD-r group (**A**,**C**) and HFD-r group (**B**,**D**) were stained with hematoxylin and eosin. Arrows indicate inflammatory cell infiltration in adipose tissue. Arrowheads indicate crown-like structures (CLSs). In intestinal sections, yellow double-headed arrows indicate villus height, and green arrows indicate crypt depth. Scale bar: 200 µm.

Histological assessment of inguinal-subcutaneous white adipose tissue and the small intestine revealed tissue morphology in *S. murinus* following FMT from differently fed donor mice. In the NCD-r group, consisting of *S. murinus* that received FMT from NCD mice, the inguinal subcutaneous white adipose tissue section showed relatively uniform adipocyte profiles (Figure 2A). In contrast, the representative adipose tissue section from the HFD-r group, which received FMT from high-fat-diet-fed mice, contained adipocytes of variable size, inflammatory cell infiltration, and crown-like structures (Figure 2B). Histological examination of the small intestinal wall showed visible villus and crypt architecture in the NCD-r group (Figure 2C), whereas in the HFD-r group, the villus and crypt appeared shorter and shallower (Figure 2D). However, this study did not perform quantitative analysis of villus height, crypt depth, and adipocyte morphology.

### 3.3. Sequencing Data Overview

**Figure 3:**
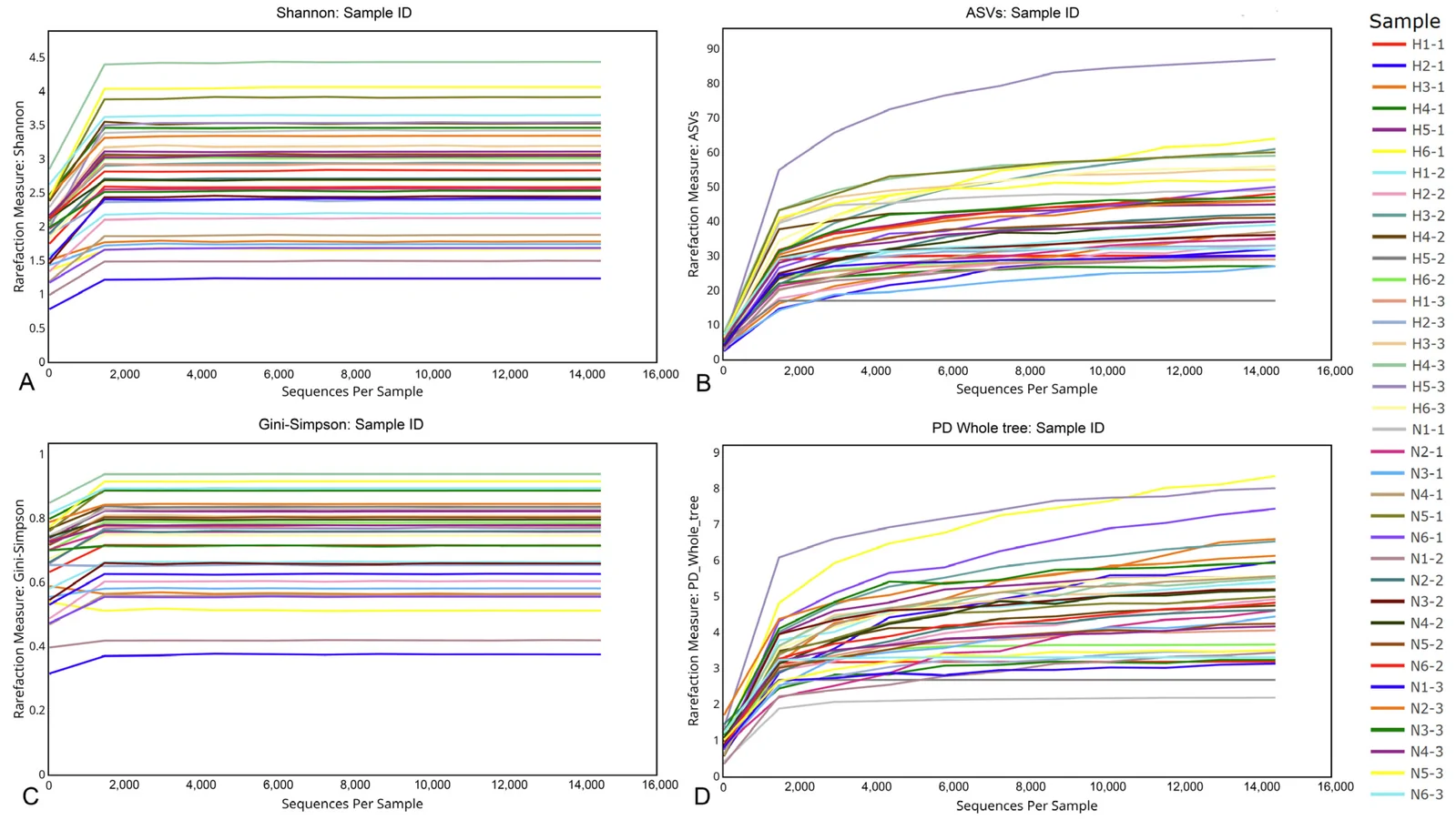
The species rarefaction curves of each experimental group. This graph represents the number of observed species under different sequence numbers extracted randomly: Shannon (**A**); ASVs (**B**); Gini–Simpson (**C**); PD_whole_tree (**D**). ASVs = Amplicon sequence variants. Gini–Simpson: The Gini–Simpson index measures the evenness of species in a community (how similar abundances are across species). Shannon: The Shannon index measures the diversity of species in a community. Phylogenetic diversity (PD_whole_tree): PD_whole_tree measures diversity by summing the total branch length of the phylogenetic tree connecting all observed taxa, thereby incorporating evolutionary relationships among species.

After filtering for chimeric and low-quality ASVs, 2,613,694 high-quality reads corresponding to 583 ASVs were identified, and 176 genera were annotated. Sequencing metrics from 16S rRNA gene sequencing (V3–V4 regions), the average coverage, and subsampling were sufficient to describe the gut bacterial community according to sequence-based rarefaction curves (Figure 3) after quality filtering.

### 3.4. Bacterial Composition and Relative Abundance

**Figure 4:**
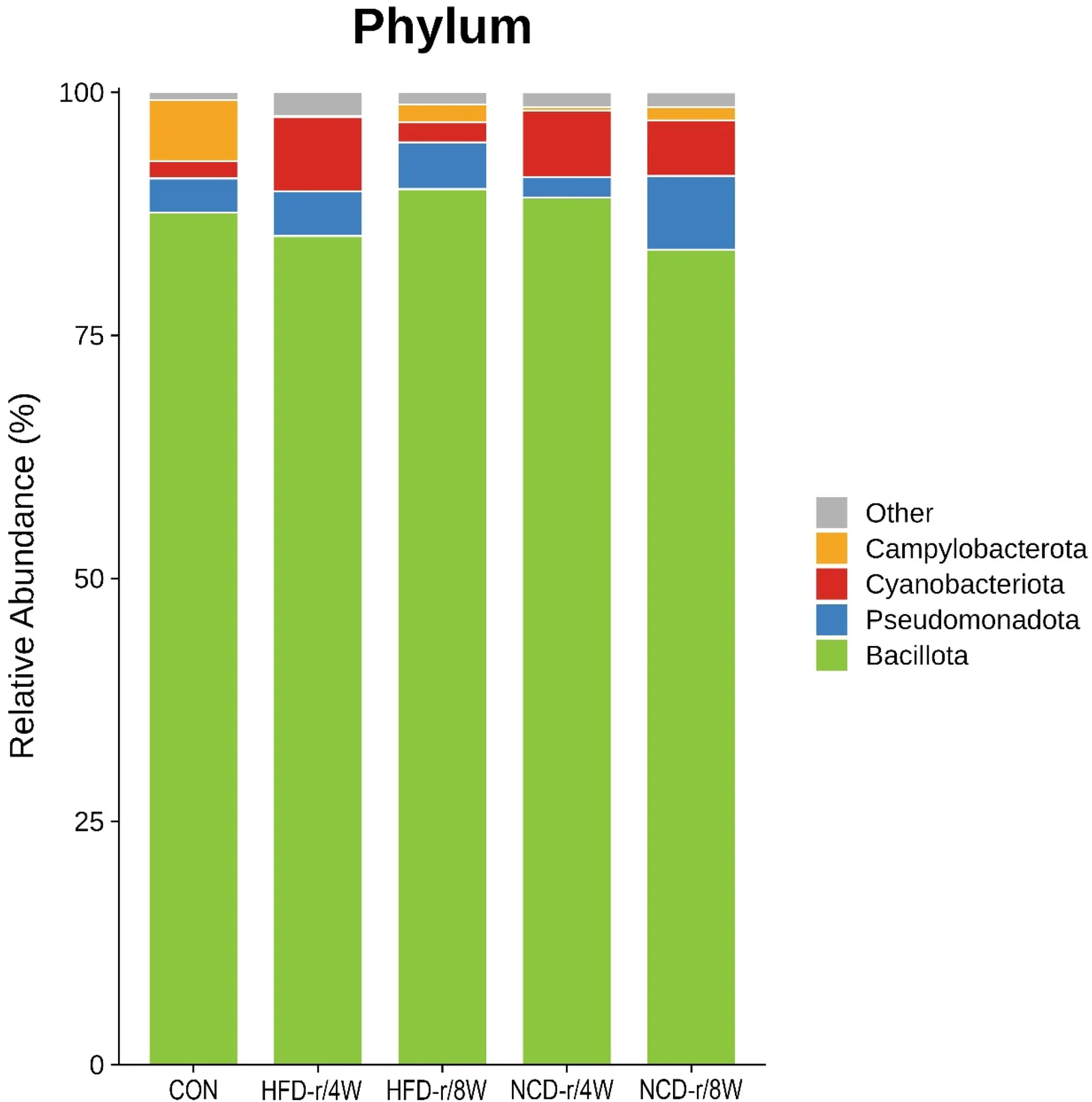
Relative abundance of the fecal bacterial microbiota at the phylum level across the experimental groups. Stacked bars show the mean relative abundance (%) of the major bacterial phyla in each group, with the remaining phyla combined as “Other”. CON represents the common pre-FMT baseline, consisting of one independent fecal sample from each of the 12 recipients. HFD-r/4W and HFD-r/8W: fecal samples collected at weeks 4 and 8 after FMT, respectively, from recipients of microbiota derived from HFD donors; NCD-r/4W and NCD-r/8W: the corresponding samples collected from recipients of microbiota derived from NCD donors.

**Figure 5:**
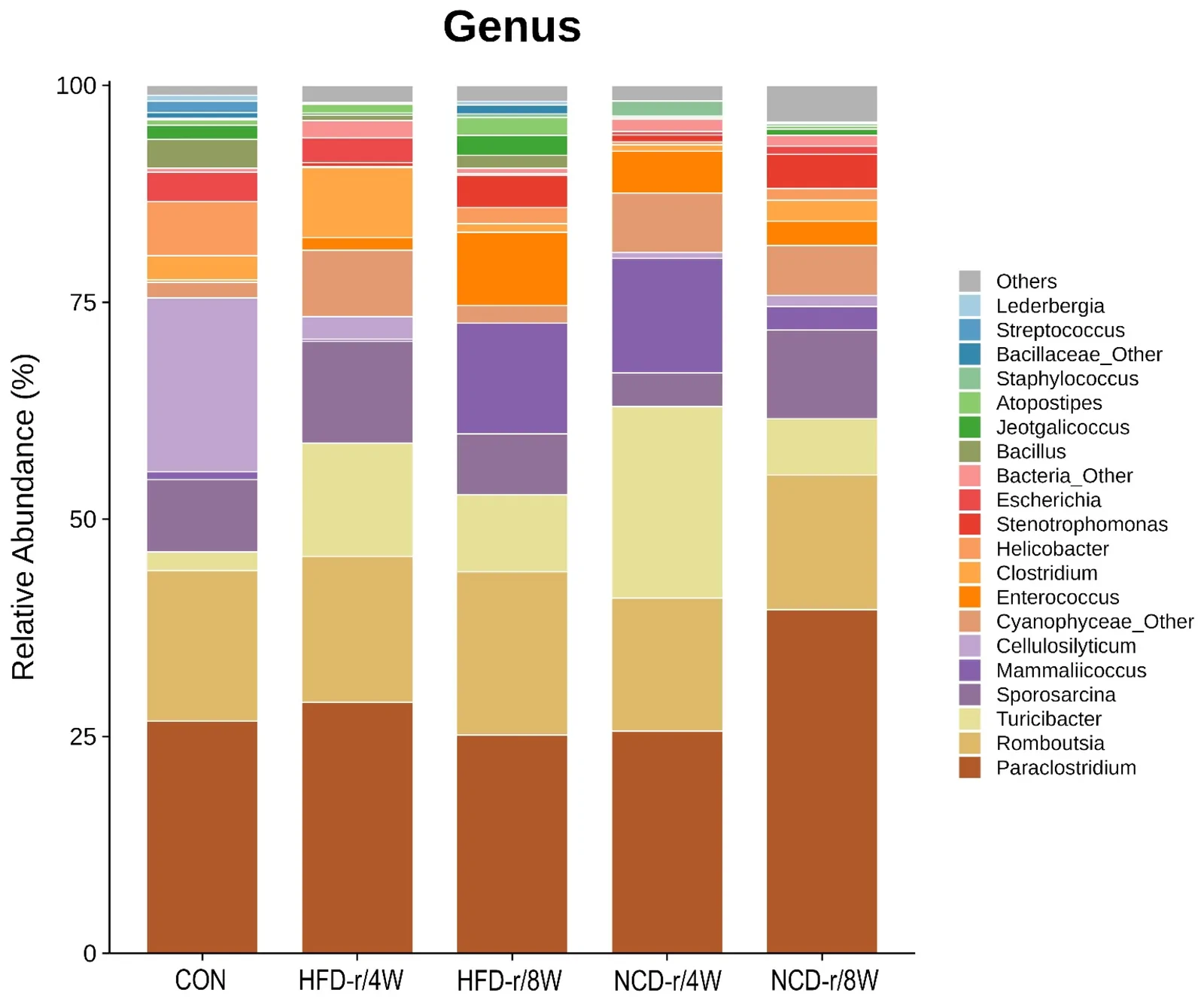
Genus-level composition of the intestinal microbiota in each experimental group. Each stacked bar represents the mean relative abundance (%) of the 20 most abundant genus-level taxa, with all remaining taxa grouped as “Others”. CON represents the common pre-FMT baseline, consisting of one independent fecal sample from each of the 12 recipients. HFD-r/4W and HFD-r/8W: fecal samples collected at weeks 4 and 8 after FMT, respectively, from recipients of microbiota derived from HFD donors; NCD-r/4W and NCD-r/8W: the corresponding samples collected from recipients of microbiota derived from NCD donors.

A total of 15 phyla, 28 classes, 55 orders, 101 families, and 176 genera were detected in the prokaryotic microbiota of all fecal samples. Based on species abundance, we selected the main phyla and genera and generated histograms to illustrate their relative abundance in each group (Figure 4 and Figure 5). These profiles describe recipient fecal samples; donor inocula were not sequenced.

At the phylum level (Figure 4), Bacillota remained the predominant component of the intestinal microbiota, accounting for 87.63% in the common pretreatment baseline (CON) and 85.23%, 90.05%, 89.19%, and 83.82% in the HFD-r/4W, HFD-r/8W, NCD-r/4W, and NCD-r/8W groups, respectively. Pseudomonadota accounted for 3.52% in the CON group and 4.59%, 4.81%, 2.10%, and 7.59% in the corresponding post-FMT groups, whereas Cyanobacteriota accounted for 1.78%, 7.64%, 2.05%, 6.84%, and 5.72%, respectively. The most pronounced and statistically supported change involved Campylobacterota, whose relative abundance decreased from 6.28% in the CON group to 0.09% in the HFD-r/4W group, 1.84% in the HFD-r/8W group, 0.34% in the NCD-r/4W group, and 1.33% in the NCD-r/8W group. A CLR-transformed constrained longitudinal data analysis showed significantly lower Campylobacterota abundance in all four post-FMT groups than in the CON group (HFD-R/4W, q < 0.001; HFD-r/8W, q = 0.027; NCD-r/4W, q < 0.001; and NCD-r/8W, q < 0.001). The sensitivity analysis using arcsine-square-root-transformed relative abundances supported this decrease in HFD-r/4W (q = 0.003), NCD-r/4W (q = 0.006), and NCD-r/8W (q = 0.038), whereas the HFD-r/8W comparison did not remain significant after FDR correction (q = 0.104). Thus, the most robust reduction in Campylobacterota occurred at 4 weeks in both treatment groups and remained detectable at 8 weeks in the NCD-r group. Changes in Bacillota, Pseudomonadota, and Cyanobacteriota were not consistently supported across the CLR and arcsine square-root analyses, and no robust differences were identified in the remaining low-abundance phyla. No robust differences were detected between the HFD-r and NCD-r groups among the dominant phyla after FDR correction. The phylum-level reduction in Campylobacterota was primarily attributable to the corresponding decrease in Helicobacter.

At the genus level (Figure 5), the intestinal microbiota was predominantly composed of *Paraclostridium* and *Romboutsia* species. The relative abundance of *Paraclostridium* was 26.77% in the CON group and 28.92%, 25.16%, 25.63%, and 39.58% in the HFD-r/4W, HFD-r/8W, NCD-r/4W, and NCD-r/8W groups, respectively. *Romboutsia* showed comparatively limited variation, accounting for 17.34%, 16.81%, 18.78%, 15.27%, and 15.53% in the corresponding groups, respectively. Several other abundant genera exhibited numerical variations. Turicibacter increased from 2.13% in CON to 13.05% in HFD-r/4W and 22.09% in NCD-r/4W, before declining to 8.89% and 6.48% in 8 weeks, respectively. Mammaliicoccus accounted for only 0.89% in the CON group but increased to 12.75% in the HFD-r/8W group and 13.19% in the NCD-r/4W group, whereas Enterococcus increased from 0.28% in the CON group to 8.44% in the HFD-r/8W group and 4.80% in the NCD-r/4W group.

The most statistically robust change involved Helicobacter, whose relative abundance decreased from 6.25% in the CON group to 0.09% in the HFD-R/4W group, 1.83% in the HFD-r/8W group, 0.34% in the NCD-r/4W group, and 1.33% in the NCD-r/8W group. The CLR-transformed constrained longitudinal data analysis showed significantly lower Helicobacter abundance in all four post-FMT groups than in the CON group (HFD-r/4W, q < 0.001; HFD-r/8W, q = 0.011; NCD-r/4W, q < 0.001; and NCD-r/8W, q < 0.001). A sensitivity analysis using arcsine-square-root-transformed relative abundances supported this decrease at 4 weeks in both the HFD-r (q = 0.012) and NCD-r (q = 0.020) groups, whereas the HFD-r/8W and NCD-r/8W comparisons were not significant after FDR correction (q = 0.156 and q = 0.078, respectively). Therefore, the strongest and most methodologically robust reduction in Helicobacter occurred at 4 weeks after FMT, consistent with the corresponding decrease in Campylobacterota at the phylum level.

Cellulosilyticum decreased from 20.07% in CON to 0.003% in HFD-r/8W and 1.27% in NCD-r/8W groups. Although these 8-week reductions were significant in the primary CLR analysis (HFD-r/8W, q < 0.001; NCD-r/8W, q = 0.047), they were not retained in the arcsine square root sensitivity analysis (q = 0.078 and q = 0.156, respectively) and were therefore considered exploratory. Changes in the remaining genera were not consistently supported across the different analytical approaches, and no genus-level differences between the HFD-r and NCD-r groups remained significant after FDR correction.

### 3.5. Alpha and Beta Diversity Patterns

**Figure 6:**
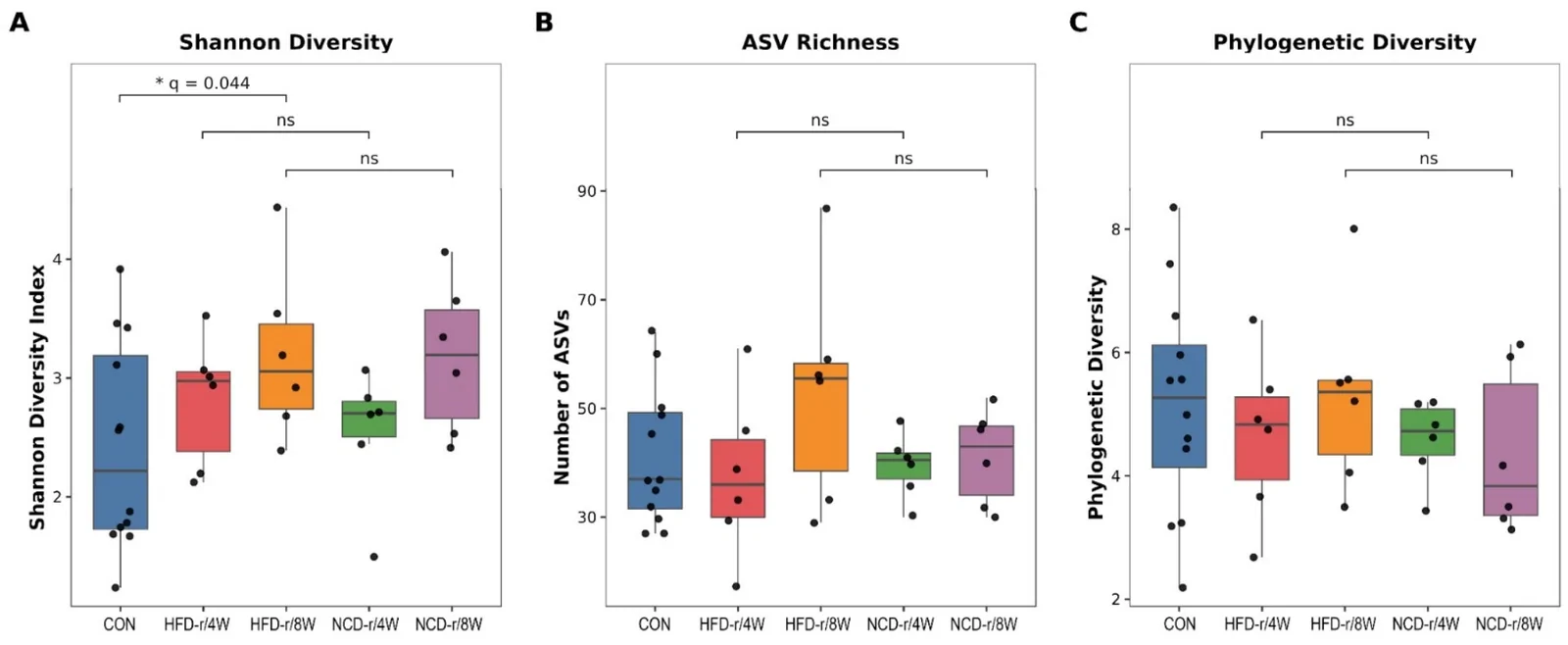
Alpha diversity of the intestinal microbiota in each experimental group. (**A**) Shannon diversity index, (**B**) observed ASV richness, and (**C**) Faith’s phylogenetic diversity. CON represents the common pretreatment baseline, comprising one fecal sample from each of the 12 recipients collected before antibiotic treatment (*n* = 12). HFD-r/4W and HFD-r/8W represent fecal samples collected at 4 and 8 weeks after FMT, respectively, from recipients of microbiota derived from HFD donors; NCD-r/4W and NCD-r/8W represent the corresponding samples collected from recipients of microbiota derived from NCD donors (*n* = 6 animals per recipient group at each post-FMT time point). Points represent individual animals. Boxes indicate the median and interquartile range, and whiskers extend to 1.5 times the interquartile range. Statistical comparisons were performed using a common-baseline constrained longitudinal data analysis (cLDA), with animal ID as a random intercept. Shannon diversity was analyzed without transformation; observed ASV richness and Faith’s phylogenetic diversity were natural-log transformed. *p*-values were adjusted using the Benjamini–Hochberg false discovery rate procedure within the prespecified alpha-diversity comparison family. Brackets labeled ns indicate nonsignificant HFD-r versus NCD-r comparisons at the same follow-up time point (q ≥ 0.05). * indicates higher Shannon diversity in HFD-r/8W than at CON (q = 0.044). All other follow-up-versus-CON comparisons were nonsignificant after FDR correction, including Shannon diversity in NCD-r/8W (q = 0.081).

Alpha diversity was evaluated using the Shannon diversity index, observed ASV richness, and Faith’s phylogenetic diversity (PD_whole_tree) (Figure 6). The CON group consisted of pre-FMT samples from all 12 recipients, whereas the HFD-r and NCD-r groups each consisted of six animals. The mean Shannon diversity, ASV richness, and Faith’s phylogenetic diversity observed in the CON group were 2.42, 41.08, and 5.17, respectively.

At 4 weeks after FMT, the mean Shannon diversity was 2.81 in the HFD-r group and 2.54 in the NCD-r group. The corresponding values for observed ASV richness were 37.50 and 39.50, while those for Faith’s phylogenetic diversity were 4.66 and 4.58, respectively. After FDR correction, none of the three indices differed significantly between the HFD-r and NCD-r groups or between either group and the CON group.

Eight weeks after FMT, the mean Shannon diversity was 3.19 in the HFD-r group and 3.17 in the NCD-r group. The observed ASV richness was 53.17 and 41.17, while Faith’s phylogenetic diversity was 5.31 and 4.36 in the HFD-r and NCD-r groups, respectively. In the common baseline constrained longitudinal data analysis, Shannon diversity in the HFD-r group was significantly higher than that in the CON group after FDR correction (q = 0.044). Shannon diversity in the NCD-r group was also numerically higher than that in the CON group, although the difference was not statistically significant after FDR correction (q = 0.081). Neither the observed ASV richness nor Faith’s phylogenetic diversity differed significantly between the groups or from the CON group. No significant differences in any of the three alpha-diversity indices were detected between the HFD-r and NCD-r groups after FDR correction.

**Figure 7:**
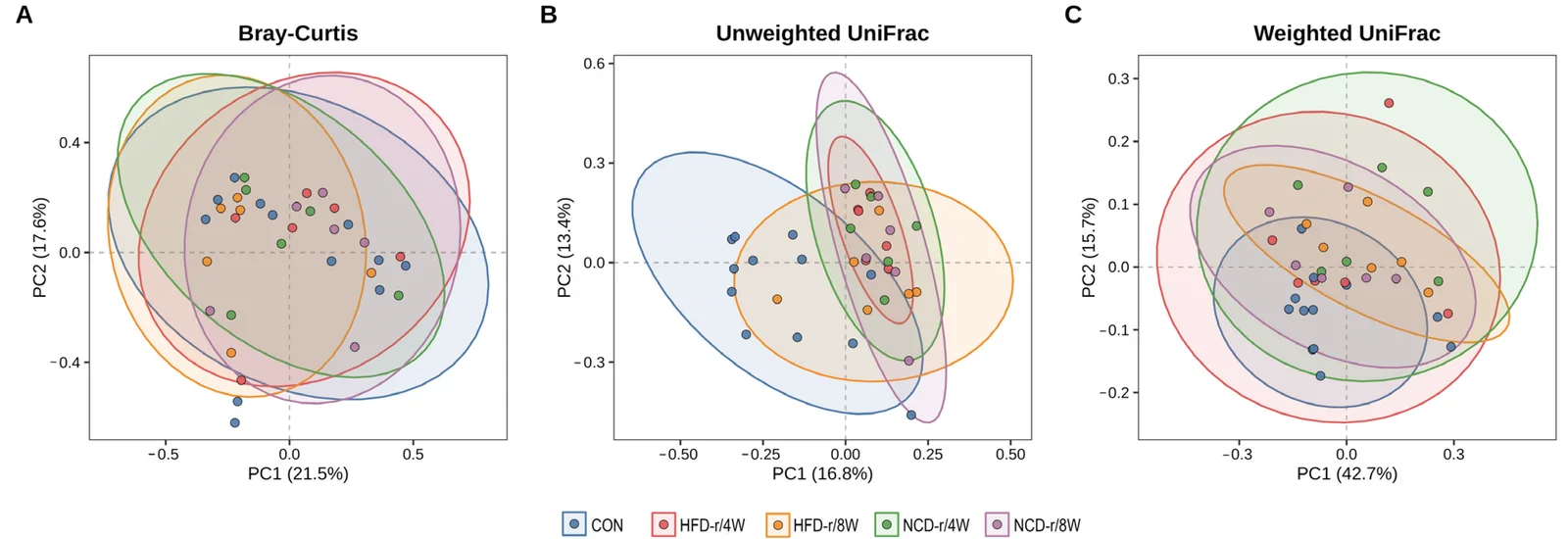
Principal coordinate analysis of intestinal microbiota beta diversity. PCoA was performed using (**A**) Bray–Curtis dissimilarity, (**B**) unweighted UniFrac distance, and (**C**) weighted UniFrac distance. Points represent individual fecal samples, and ellipses summarize the within-group covariance across the first two principal coordinates. Percentages shown on the axes indicate the proportions of variation explained. CON represents the common pre-FMT baseline, comprising one independent fecal sample from each of the 12 recipients. HFD-r/4W and HFD-r/8W: fecal samples collected at 4 and 8 weeks after FMT, respectively, from recipients of microbiota derived from HFD donors; NCD-r/4W and NCD-r/8W: the corresponding samples collected from recipients of microbiota derived from NCD donors.

Beta diversity was evaluated using a principal coordinate analysis (PCoA) based on Bray–Curtis, unweighted UniFrac, and weighted UniFrac distances (Figure 7). The first two principal coordinates jointly explained 39.17%, 30.18%, and 58.43% of the total variation in the three analyses. Considerable overlap among the experimental groups was observed in all three ordination plots. Although the unweighted UniFrac analysis suggested some displacement of the post-FMT samples from the CON samples, the HFD-r and NCD-r groups remained extensively overlapped with each other. At 8 weeks, the group centroids showed a modest displacement in the Bray–Curtis and weighted UniFrac ordinations, suggesting weak compositional differentiation between the HFD-r and NCD-r groups; however, this visual pattern was not evident across all three distance measurements.

Restricted PERMANOVA detected no significant differences between the HFD-r and NCD-r groups at either 4 or 8 weeks after FDR correction. No overall differences were detected between the two groups during the post-FMT period. Comparisons of the post-FMT samples with their corresponding paired baseline samples were also not significant after FDR correction. PERMDISP detected no significant differences in multivariate dispersion. Test statistics, effect sizes (R^2^), *p*-values, and adjusted q-values are reported in Supplementary Table S1.

### 3.6. Bacterial Composition Difference Analysis

**Figure 8:**
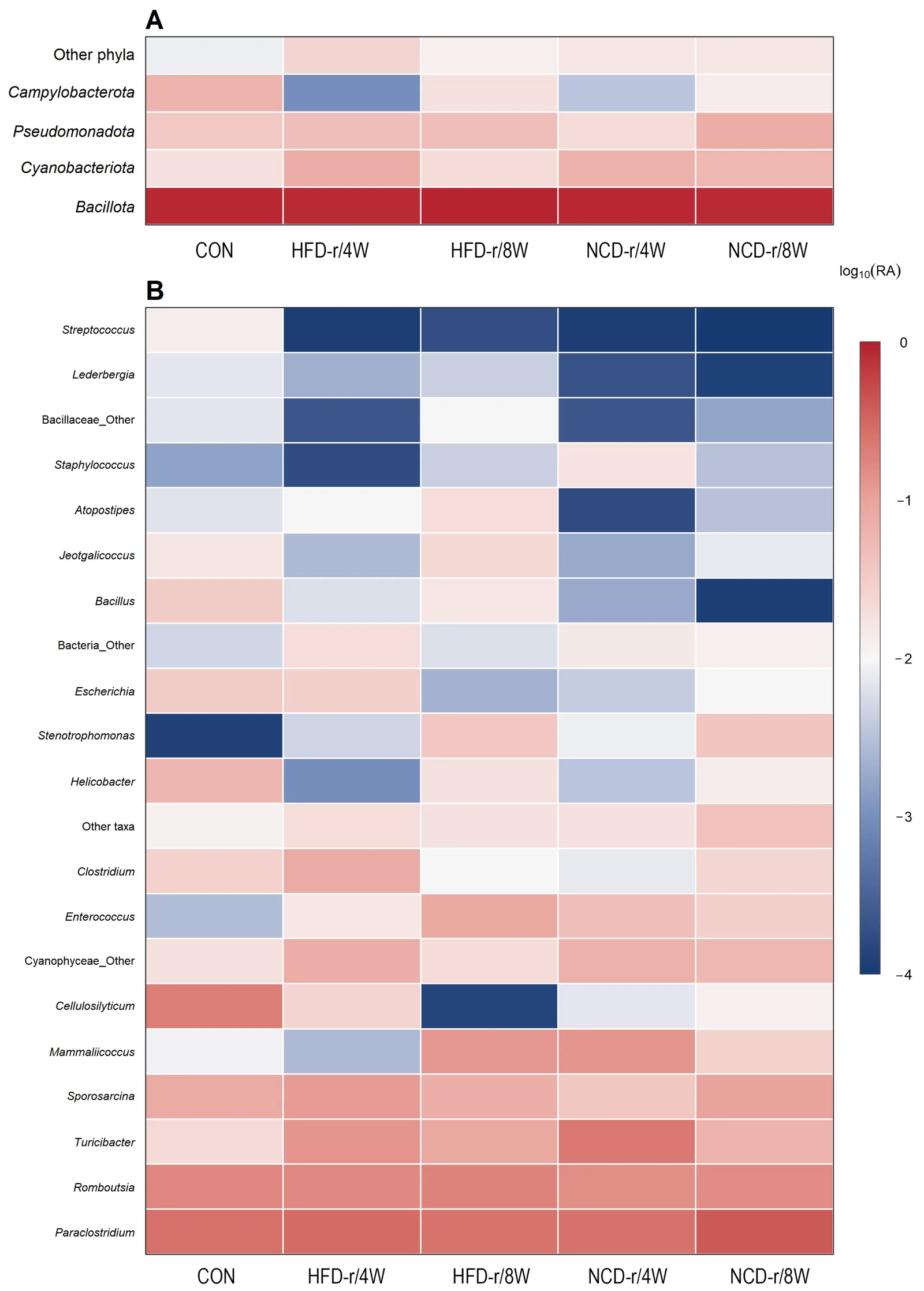
Heatmap of mean bacterial relative abundance at the phylum and genus levels across the experimental groups. (**A**) The four most abundant named phyla and the combined abundance of all remaining phyla (“Other phyla”). (**B**) The 20 most abundant genus-level taxonomic categories and the combined abundance of all remaining taxa (“Other taxa”). Taxa were selected according to their overall mean abundance across all samples. Each cell represents the group mean relative abundance transformed as log10(RA + 10^−4^), where RA is expressed as a proportion. The shared color scale ranges from blue (lower relative abundance) to red (higher relative abundance). CON represents the common pre-FMT baseline, comprising one independent fecal sample from each of the 12 recipients. HFD-r/4W and HFD-r/8W: fecal samples collected at 4 and 8 weeks after FMT, respectively, from recipients of microbiota derived from HFD donors; NCD-r/4W and NCD-r/8W: the corresponding samples collected from recipients of microbiota derived from NCD donors.

Heatmaps of group means provided a complementary overview of the bacterial composition at the phylum and genus levels (Figure 8). Bacillota remained the predominant phylum across all groups, whereas *Paraclostridium* and *Romboutsia* were consistently among the most abundant genus-level taxa. The lower abundance of Campylobacterota and its major constituent genus Helicobacter in post-FMT samples, particularly at 4 weeks in both the HFD-r and NCD-r groups, was consistent with the preceding abundance analyses. Although several lower-abundance taxa varied among groups, these patterns were heterogeneous, and no coherent separation between HFD-r and NCD-r recipients was evident at any post-FMT time point.

## 4. Discussion

This study investigated whether gut microbiota from HFD donors could generate persistent microbial and host phenotypes in *S. murinus*. Over an 8-week observation period, no statistically significant between-group differences in body weight or glucose tolerance were observed in the recipient *S. murinus*. However, representative adipose and small intestinal sections showed qualitative variation between recipient images. Fecal microbial communities showed extensive overlap across donor groups: after false discovery rate correction, no clear differences were detected between the HFD-r and NCD-r groups regarding alpha diversity, beta diversity, or the relative abundance of individual phyla or genera. However, the phylum Campylobacterota and its major constituent genus *Helicobacter* decreased in both recipient groups. These results describe similar recipient fecal community patterns under the two donor-source conditions; recipient-associated ecological constraints remain a possible explanation.

The absence of body weight or glucose tolerance divergence contrasts with transplantation studies in permissive recipient systems in which obesity-associated microbiota transmit metabolic traits [9]. However, the absence of significant between-group metabolic differences does not exclude more localized responses. Adipose tissue exhibited “crown-like structures” and heterogeneous adipocyte morphology in recipient animals receiving microbiota associated with a high-fat diet, while representative small intestinal sections showed variation in villus and crypt appearance. These descriptive findings warrant quantitative assessment and should not be interpreted as direct evidence of macrophage-mediated inflammation or impaired barrier permeability, as the study did not assess histomorphometric parameters, inflammatory mediators, tight-junction proteins, and functional permeability. The limited systemic metabolic response may also reflect the natural obesity-resistant physiology previously described in *S. murinus* [18]. Accordingly, histological observations identified a tissue-level signal that warrants quantitative confirmation rather than demonstrating the transmission of a complete obesity phenotype.

Community-level analyses also provided little evidence of a donor diet-specific microbiota response. Shannon diversity was higher in the HFD-r group at 8 weeks than in the common baseline group after FDR correction; however, the NCD-r group changed in the same direction, and no HFD-r and NCD-r differences were detected. Observed ASV richness and Faith’s phylogenetic diversity remained comparable among the groups. In parallel, the Bray–Curtis, unweighted UniFrac, and weighted UniFrac ordinations showed extensive overlap, and the restricted PERMANOVA did not detect significant HFD-r vs. NCD-r differences at either post-FMT time point or over the post-FMT period as a whole. PERMDISP was also not significant. Thus, the modest ordination displacements and isolated Shannon results should not be interpreted as evidence that the HFD-donor microbiota produced a distinct recipient community. Because every recipient underwent antibiotic conditioning and FMT, changes from the common pretreatment baseline may reflect the combined effects of microbial depletion, re-colonization, maturation, and transplantation, rather than an effect that can be assigned to FMT alone [22,36].

The results of taxonomic analyses further support this interpretation. Across all groups, Bacillota was the predominant phylum, consistent with previous reports that the gut microbiota of *S. murinus* is enriched with Bacillota [17,37]. At the taxonomic level, the most consistent change observed at week 4 was a decrease in the abundance of the phylum Campylobacterota and the genus *Helicobacter* in the HFD-r and NCD-r groups. The corresponding 8-week comparisons were less consistent across analytical transformations, particularly at the genus level, and no robust differences were detected between the HFD-r and NCD-r groups. Previous studies have identified *Helicobacter* as an abundant genus in the *S. murinus* microbiota [37]. Thus, the concurrent decline in its abundance signifies a substantial ecological shift in community structure, marking a state distinct from the pretreatment baseline. However, genus-level 16S rRNA profiling cannot elucidate the specific species, strains, virulence potential, or functional consequences associated with this change. Therefore, the most appropriate interpretation is to view the reduction in *Helicobacter* abundance as a shared feature of the microbiota restructuring process following antibiotic pretreatment and FMT, rather than as a beneficial or HFD-specific response to diet. Changes in the abundance of other genera were not consistently confirmed in either the primary or sensitivity analyses, and further investigation is required to confirm these findings.

“Recipient filtering” provides a possible ecological framework for interpreting the similar recipient community patterns. Microbiota colonization is not merely a process of donor microbiota replacing the recipient’s indigenous flora; studies on fecal microbiota transplantation resolved at the strain level indicate that donor and recipient strains can coexist, with colonization outcomes determined by the baseline microbiota, ecological compatibility, and available niches [11,12,13,14]. Reciprocal transplantation experiments between distantly related vertebrate hosts have further confirmed that the recipient’s gut habitat can redirect the relative abundance of exogenous microbial lineages, driving them to evolve toward a microbiota structure characteristic of the recipient [38]. In this study, the donor material originated from mice fed either HFD or NCD, while the recipients were insectivorous shrews fed an identical diet. The gut microbiota of the recipient groups showed a high degree of overlap and maintained a community structure dominated by Bacillota, along with a decrease in the abundance of Campylobacterota/*Helicobacter*. These patterns are compatible with recipient-associated ecological constraints, although the common recipient diet and pretreatment may also contribute.

The biological characteristics of *S. murinus* make this filtering hypothesis plausible. Its intestinal tract is short and anatomically simple, lacking a cecum, and differs substantially from the gastrointestinal habitat of mice. Its native microbiota is also quite unique among laboratory mammals, characterized by the dominance of Bacillota and a low abundance of Bacteroidota [17,37]. Furthermore, previous studies have shown that *S. murinus* is naturally resistant to diet-induced obesity [18], and *S. murinus* microbiota can reshape the gut ecology of HFD-induced obese mice [19], emphasizing that transplantation outcomes depend on the recipient environment and may not be symmetrical across host species. Because the donor inocula were not sequenced, initial donor community differences and the persistence of donor-derived organisms in recipients could not be assessed; recipient-specific filtering therefore remains a hypothesis requiring paired donor–recipient analysis. Consequently, it is difficult to ascertain whether the lack of significant differences between recipient groups stems from host-specific microbial filtering, insufficient microbial colonization, or simply the fact that the initial donor microbial communities were already very similar to begin with.

The discrepancy observed between the taxonomic composition of the fecal microbiota and tissue morphology warrants careful consideration. Similarity in abundance at the phylum level does not guarantee consistency in strain composition or the host physiological function; even when total bacterial loads and broad phylum profiles appear similar, colonization of host-specific versus non-host-specific microbiota can still lead to divergent intestinal immune outcomes [39]. In the present study, HFD-r and NCD-r derived communities may have already differed transiently before the first 4-week sampling point, at the strain or gene content level, or in metabolite production, without generating persistent separation in fecal 16S sequencing profiles. Furthermore, fecal samples may not fully reflect the microbiota composition of the small intestinal mucosal regions evaluated histologically. Alternatively, qualitative differences at the tissue level may reflect inter-individual variation rather than reproducible effects mediated by microbiota. To elucidate these possibilities, it is necessary to conduct a paired analysis of the donor inoculum and recipient using shotgun metagenomics or strain-resolution techniques, integrated with metabolomics, an analysis of mucosa-associated microbiota, quantitative histology, and measurement of markers related to the inflammatory and barrier functions.

However, the reliability of these conclusions is constrained by several design limitations. No a priori sample-size or power calculation was performed. The choice of six recipients per group was based on animal availability due to breeding difficulties and an earlier small-scale FMT study using six mouse recipients per group [19]. The small number of recipients (*n* = 6 per donor diet group) may have limited the statistical power to detect subtle effects and increased susceptibility to inter-individual variation. Nonsignificant between-group differences do not establish biological equivalence. The CON group served as a pretreatment baseline rather than a concurrent untreated control, and the study lacked groups receiving only antibiotics, sham FMT, or no treatment; consequently, it is impossible to attribute common post-treatment changes specifically to antibiotic recovery, aging, or the FMT effect. Specifically, the body-weight comparisons do not establish an FMT effect or a specific change from pre-FMT weight. Without pretreatment IPGTT measurements or a concurrent untreated control, the glucose-tolerance results cannot establish changes from baseline or an FMT effect. Because each donor diet group was housed in a single cage and the two cages were maintained in separate compartments, donor diet effects could not be distinguished from cage or compartment effects. The HFD and NCD also differed in ingredients beyond fat content. The study did not profile the donor inocula or measure absolute bacterial loads, and fecal 16S sequencing failed to provide strain-level or functional resolution of the data. The performance of post-FMT sampling only at weeks 4 and 8 may have missed instances of early transient colonization, while the endpoint histological analysis was merely qualitative, lacking quantitative morphometry, blinded scoring, or molecular validation. Therefore, future studies should employ replicated donor-cage setups and appropriate antibiotic or sham treatment controls, sequence each paired donor inoculum, perform high-frequency sampling of recipients during the initial weeks, and integrate strain tracking, metabolite profiling, mucosal and fecal sampling, a quantitative histomorphometric analysis, and assessments of the barrier function and immune parameters.

## 5. Conclusions

In this study, no significant differences in fecal microbial composition, body weight, or glucose tolerance were detected between HFD-r and NCD-r in *S. murinus* over 8 weeks, although qualitative variation was observed in representative tissue sections. The overlap of the HFD-r and NCD-r communities is compatible with the hypothesis that the distinctive intestinal environment of *S. murinus* may constrain the persistence of donor diet-associated microbial signals following cross-species FMT. Further work is needed to evaluate *S. murinus* as a candidate model for investigating recipient-dependent constraints on microbiota engraftment. However, paired donor–recipient strain tracking, replicated donor cages, appropriate controls, and functional validation are necessary to establish this potential.

## Data Availability

The 36 samples have been registered in NCBI BioSample (accession numbers: SAMN64336127–SAMN64336162). The processed ASV abundance table is provided in Supplementary Data S1. Associated sample metadata have been submitted to NCBI BioSample. Raw sequencing data have been deposited in the NCBI Sequence Read Archive (SRA) (BioProject accession number: PRJNA1548240).

## Supplementary Materials

The following supporting information can be downloaded at: https://www.mdpi.com/article/10.3390/nu18213626/s1, Figure S1: The body weight curves and IPGTT results of the HFD-r group and NCD-r group; Table S1: Exact PERMANOVA and PERMDISP results; Data S1: Processed ASV abundance table.
