# Supplementary material for: Remodeling and Alteration of the Gut Microbial Community in Suncus murinus Receiving Fecal Microbiota Transplantations from Mice with High-Fat Diet-Induced Obesity
Source: Nutrients. 2026 Oct 31;18(21):3626. doi: 10.3390/nu18213626 (PMC13765305; doi:10.3390/nu18213626)
Supplement: Supplementary file 1 [file nutrients-18-03626-s001.zip › TABLE S1.pdf]

Table S1. Exact PERMANOVA and PERMDISP results

Index	Shannon	Gini-Simpson	PD_whole_tree	ASVs	Good's Coverage
H1	2.3067 ± 0.8812	0.6451 ± 0.1962	5.4786 ± 2.0018	39.17 ± 13.70	1.0000 ± 0.0000
H2	2.8101 ± 0.5444	0.7542 ± 0.0976	4.6559 ± 1.3439	37.50 ± 15.10	1.0000 ± 0.0000
H3	3.1929 ± 0.7280	0.7907 ± 0.0943	5.3066 ± 1.5657	53.17 ± 20.88	1.0000 ± 0.0000
N1	2.5341 ± 0.9464	0.6960 ± 0.1499	4.8699 ± 1.7052	43.00 ± 12.08	1.0000 ± 0.0000
N2	2.5409 ± 0.5509	0.7019 ± 0.1484	4.5813 ± 0.6656	39.50 ± 6.06	1.0000 ± 0.0000
N3	3.1737 ± 0.6405	0.7953 ± 0.1115	4.3614 ± 1.3412	41.17 ± 8.77	1.0000 ± 0.0000

ASVs: amplicon sequence variants. Shannon: The Shannon index measures the diversity of species in a community. Gini-Simpson: The Gini-Simpson index measures the evenness of species in a community (how similar abundances are across species). Phylogenetic diversity (PD_whole_tree): PD_whole_tree measures diversity by summing the total branch length of the phylogenetic tree connecting all observed taxa, thereby incorporating evolutionary relationships among species. Good's Coverage, Coverage is calculated as $C=1-(s/n)$.

H1–H3: baseline, 4-week, and 8-week fecal samples from the HFD group. N1–N3: baseline, 4-week, and 8-week fecal samples from the NCD group.
